# Supplementary material for: Chromothripsis during telomere crisis is independent of NHEJ, and consistent with a replicative origin
Source: Genome Res. 2019 May;29(5):737–49. doi: 10.1101/gr.240705.118 (PMC6499312; doi:10.1101/gr.240705.118)
Supplement: Supplemental Material [file supp_gr.240705.118_Supplemental_file_1.zip › contigs/annotated_contigs/DB50/contig.3.DB50_length_682_mean_cov_10.3372434018.docx]

**DB50_length_682_mean_cov_10.3372434018**

```
ATTCTCATAGCAAAGCAAGAGAAACCAAGAGACTAGTACCATGTAGTAAATGAACATCATGCCAGTTCAAATAAACACAGGCTCAGAAG
 >chr4:109398536-109398712 + E=4e-94
TGAATGTAAGAATGAGAACAAATAACTAAAATAAGGCACACTGGGCTAAATAAACTCAAGTCCATAATGAGAATGGAGGC|CAGTTAG|
                                                                                 >chr4:1
ATGAAATTGATCTTTCTATGTTTTCCTTCTTCCAGATCTCCACTCTTCCCATTCTGGCTAACTGGCCTCCATTCTCATTATGGACTTGA
09398661-109398776 - E=2e-57 p=1e-02
GTTTATTTAGCCCAGTG|TG|GCCTCAGAATAGTGCTCAGGAAAAGCGACACATTTGAGTAAGGGAATTGGTCAATCTTCCCATTACAA
                  >chr4:109399673-109400084 + E=1e-212
ATATCTTTATGACAACTGGAAATTTTCGTTAAAATTATTTTAAAACCTTCTTAATTATTTCTAAAAAAAAAAAAGATACAGAACAGGTG

TAGGTAAACTACTGCCAGCAGGCCAGTTTTGGCCCTGAGTCTGTTTTTGAAAGGAAAGTTCATTGGGATGCAGCTATGCTCATTCATTT

ATGTATTGTCTGTGGCTGTTTTTGCACTATAACAGCAGAACTGAGTAACTGCAAGAAAGACCCTACGACCAACAAAACTAAAAATATTA

CTATCTGTCTCTTTACATAAAAAGTTTCCCAGCAGCTGATATAGCCCAACTGTTTCAAGCACT
```
